# Citizens' conceptions of the genome: Related values and practical implications in a citizen forum on the use of genomic information

**DOI:** 10.1111/hex.13187

**Published:** 2021-01-16

**Authors:** Chloé Mayeur, Wannes van Hoof

**Affiliations:** ^1^ Department of Public Health and Monitoring Cancer Center, Sciensano Brussels Belgium

**Keywords:** common good, ethics, genomics, individual property, privacy, public engagement

## Abstract

**Background:**

The development of large data sets, including genomic data, coupled with rapid advances in personalized medicine where citizens increasingly face complex choices about the use of their genomic information implies that citizens are essential stakeholders in genomics. They should be engaged in the ethical, legal and societal issues to produce a framework that fosters trust and allows them to guide the technology based on their values.

**Objective:**

This article highlights that citizens' conceptions of the human genome inform about and make sense of their main values regarding the use of genomic information, which is critical for policymakers, experts and stakeholders to understand to maintain the public support in genomics.

**Method:**

Through an inductive thematic approach, we reanalysed data collected for the Belgian citizen forum, which aimed to produce recommendations for the Ministry of Public Health and other stakeholders.

**Results:**

Citizens expressed four conceptions of the genome that determined which uses of genomic information they supported: the most intimate part of individuals; ‘I am more than my genome’; the individual's property vs the common good; and uncertainty and fear.

**Conclusion:**

Diversity in their conceptions reveals remaining conflicts of values among citizens, mainly regarding a conception of the genome as an individual property or a common good. However, despite differing conceptions, shared values emerged such as solidarity, privacy, no genetic discrimination and the right to an open future, where individual and common interests coexist.

**Patient or public contribution:**

The panel of the citizen forum consisted of 32 citizens.

## 1 INTRODUCTION

Rapid advances in personalized medicine and genomic technologies raise new questions and issues from conception (eg prenatal and neonatal screening) to adulthood (eg genealogy, carrier screening, precision medicine).^1^ Consequently, citizens increasingly have to make complex and personal choices about the use of their genomic information. To support the implementation of these technologies, governments are spending many public funds worldwide as well.^2^ Additionally, the successful deployment of genomic data for preventive and medical purposes requires the development of a large representative data set, including long‐term health and environmental data of patients and healthy individuals. Hence, the use of genomic information and related technologies calls for the support of the public. Public engagement on the ethical, legal and societal issues (ELSI) in genomics allows citizens to guide the technology based on their values and principles, resulting in a framework that fosters trust in the use of genomic information in society.

Public engagement on ELSI in genomics can take many forms. It often involves questioning patients, professionals and research participants through surveys and interviews (eg Dheensa et al^3^; Middleton et al^4^). Some studies have also described attitudes towards genomics in the general public.^5^, ^6^ These studies are very informative, but most of them remain descriptive and do not explicitly translate the results into policy and regulatory recommendations. Recently, several countries have decided to tackle the issue of developing a policy framework for genomics by using deliberative processes with citizens. In France, the public, experts and other stakeholders were consulted as part of the review of the French law on bioethics with a specific interest in genomics.^7^ Genomics England reported on their public engagement efforts regarding genomics that citizens call for a new social contract in health care.^8^ Within the European Horizon 2020 programme, a stakeholder involved ethics project (SIENNA) was launched with genomics as one of the core subjects.^9^

In Belgium, Sciensano and the King Baudouin Foundation organized a citizen forum at the request of the Minister of Public Health. A panel of 32 citizens considered the place of the human genome in society and formulated recommendations on the use of genomic information. The Minister and relevant stakeholders listened to and discussed with the citizens about their recommendations.

The qualitative analysis of transcribed discussions among citizens made it clear that citizens' conceptions of the genome inform some of their main values regarding the use of genomic information. It is critical for policymakers, experts and stakeholders to understand these values and where they come to maintain the public support in genomics.

## 2 METHOD

### 2.1 The citizen forum as a method

A citizen forum is an internationally approved method where a small group of selected citizens, generally 16‐32 individuals, debate and reflect in‐depth on complex and controversial societal issues.^10^, ^11^ It is a way to create practical policy input with involvement from citizens, experts, stakeholders and policymakers.^13^

### 2.2 Participant selection and recruitment

With the help of 10 non‐profit civil organizations, targeted media calls and the Bpact online recruitment database, 492 citizens responded to an initial open call to participate in the citizen forum. Candidates received an online questionnaire with socio‐demographic questions, and questions about their motivation to participate and about their knowledge of the topic. The goal was to form the most diverse citizen panel possible to generate high‐quality discussions. With this in mind, the King Baudouin Foundation and the independent research agency Indiville selected 160 citizens who answered the online questionnaire, according to criteria such as age, gender, language, level of education, work and family situation (Table 1). People with severe genetic disease and professionals in the field of genomics—doctors, researchers or experts—were excluded. From these 160 citizens, 32 were selected to form the most diverse panel possible. Participation was voluntary—three participants did not come to the last weekend—and they were paid a small fee to reimburse travel costs.

**TABLE 1:** The citizen panel (N = 32)

Criteria of selection	Number of participants per criterion
Gender	
Men	16
Women	16
Language	
Dutch	16
French	16
Age	
18‐25	5
26‐45	12
46‐65	15
Work situation	
Employee	11
Public servant	3
Self‐employed	2
Pensioner	4
Work incapacity/disability	1
Voluntary housewife/house husband	3
Jobseeker	4
Student	4
Level of education	
Primary education	2
Technical education	1
Vocational education	1
Secondary education	8
Artistic education	1
Higher education	19
Family situation	
Alone	7
Cohabitation with spouse/children/parents/parents‐in‐law	23
Community housing (eg student housing, nursing home)	2
With children	20
Without children	12
Connection to the health sector	
Is or was professionally active	3
No professional connection	29

### 2.3 The Belgian citizen forum

**FIGURE 1:**
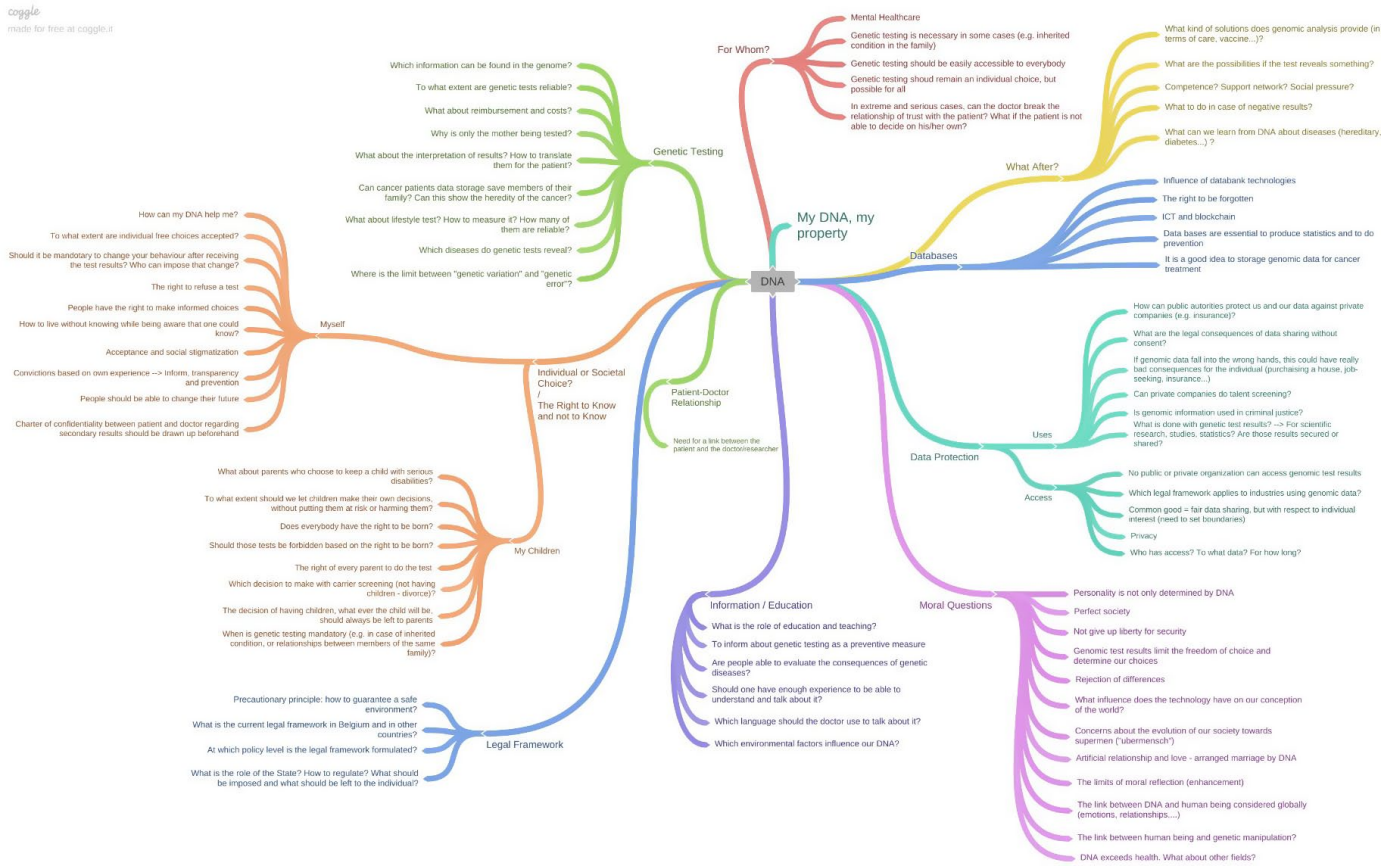
The mind map, which summarizes the main themes, their related issues and values identified by participants

**FIGURE 2:**
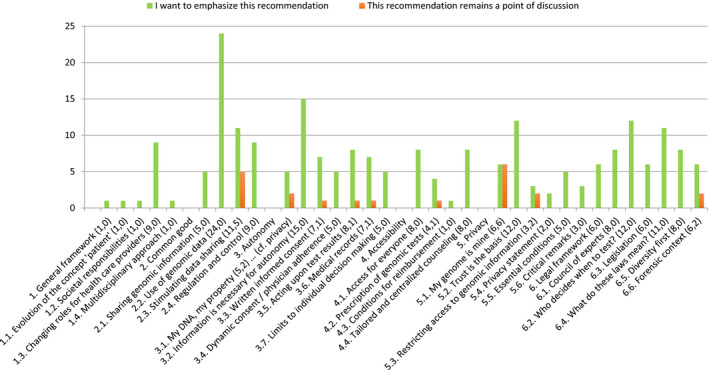
Each citizen received three red tokens to indicate the recommendations where areas of tension remained and three green tokens for the recommendations they supported the most. There was no obligation to use all the tokens

During 3 weekends, this panel debated and reflected on ELSI surrounding the use of genomic information. The starting point of the discussion was an informative booklet, sent to participants in advance. By use of nine practical cases, it explained how genomics might influence people's lives and entail complex individual and collective choices (personalized medicine for cancer treatment; informing the family; carrier screening; prenatal and neonatal genetic testing; secondary findings; managing databanks; direct‐to‐consumer testing; and non‐medical screening—eg behavioural traits and talents).^14^ A team of facilitators, translators and 14 resource persons with different expertise related to the topic (law, genetics, biobank, ethics, patient associations, et cetera) helped the citizens formulate balanced and well‐informed opinions by informing them, answering their questions and challenging them through various deliberation methods. Among others, we used the journalism method (in groups of four, each citizen had to interview the rest of the group like a journalist about one case of their choosing in the booklet) and role‐plays (citizens simulated a discussion between a patient, a doctor‐researcher and a patient organization about data sharing; and an employer asking his employee to access his genomic data, with the intervention of the trade union). Those methods foster critical attitudes, individual and collective reflection, facilitate a better mutual understanding, pinpoint remaining areas of tension and highlight opportunities for improvements on current practices. Those exercises led to the creation of a mind map, which summarized the main themes, their related issues and values identified by participants (Figure 1). The second weekend aimed at deepening all themes; by interacting with experts on issues, citizens indicated they needed more information to formulate their opinion. This allowed them to voice their values correctly and to write the final recommendations during the last weekend. Citizens criticized and improved their recommendations before indicating which ones they prioritized and the ones that still included areas of tension (Figure 2).

An advisory committee supervised the quality of the informative background material and supported the selection of resource persons. Two independent researchers assessed the quality of the process. They published their assessment in a separate report.^15^ Throughout the citizen forum, particular attention was paid to transparency and to give participants an active role both on content and methodology. For instance, participants chose the areas of expertise of the resource persons they needed, they reviewed and criticized the content of the deliberations thanks to detailed summary reports we sent them after each weekend, and we took their remarks into account on the final report, which they approved.^16^ This article is a qualitative analysis of a public engagement initiative, not a qualitative study per se. Nevertheless, the citizen forum was conducted in a way that followed the Declaration of Helsinki: participation was voluntary, participants could refuse to engage in discussions at any point and consented to the recording of all discussions.

### 2.4 Stakeholder involvement

Stakeholders were involved in all stages of the process. The citizen forum started with an issue framing workshop, where more than 50 Belgian and international experts and policymakers framed and identified the ELSI that citizens should discuss.^17^ An advisory committee composed of experts from various fields continually monitored and mentored the project. During the 3 weekends, several experts were invited to engage in the discussions. After the citizen forum, we organized a stakeholder workshop where experts and stakeholders analysed the implementation of the citizens’ recommendations into the Belgian health‐care system.^13^

### 2.5 Data analysis

All discussions, both between citizens and experts and among citizens, were audio‐recorded and transcribed. As participants were Dutch‐ and French‐speaking, simultaneous translation was provided in all plenary and some subgroup sessions. We ensured that all researchers performing the data analysis were fluent in both languages and that at least one researcher was a native speaker.

Results of the general inductive thematic analysis of citizens' recommendations have been written up in a report that answers to the research question ‘How should we deal with genomic information in society?’.^18^ We started with an open‐coding process.^19^ One researcher coded the transcriptions manually, another checked the codes and entered them in Nvivo, while a third researcher resolved disparities between interpretations. The codes were kept close to the original data, which allowed themes to emerge naturally. These themes were grouped in bigger themes with careful consideration for interconnectedness by two researchers who were constantly debating alternative interpretations. We stayed vigilant to focus sufficiently on significant disagreements and divergent opinions.

While performing this first analysis, one central theme—the conceptions of the genome—evolved into a separate research question because we realized how strong these conceptions influence the citizens' recommendations and make sense of their values. The new research question was ‘What do citizens’ conceptions of the genome say about the uses of genomic information supported or disapproved in society?'. In the light of this new research question, we decided to re‐analyse in‐depth the original codes related to the conceptions of the genome, and to restructure the thematic analysis to produce the present article.

The reports published during the citizen forum are descriptive and aim to communicate the policy recommendations produced by citizens. They do not include a qualitative analysis.

## 3 RESULTS

The inductive thematic analysis of the discussions among participants showed four conceptions of the human genome:

1. The genome is the most intimate part of individuals that warrants protection to avoid a breach of privacy.
2. The genome does not tell the whole story about individuals, or ‘I am more than my genome’.
3. The individual's property vs the common good.
4. The genome implies uncertainty and fear.

### 3.1 The most intimate part of individuals

All citizens agreed that their genome defines who they are since it is unique to each individual. Citizens compared the genome to a barcode that renders individuals always identifiable and talked about it as an ‘internal identity card’. They considered the information that can be extracted from it to be a part of an individual's ‘intimacy’, that is private life. By privacy, citizens meant all information individuals want to keep confidential because its disclosure could negatively affect their lives by leading to rejection, negative judgement or discrimination. For example, the disclosure of genetic diseases may change the way relatives, employers or society at large consider and treat someone as a person.

In a society that collects and shares a large amount of personal data daily, participants considered privacy protection to be of great importance in genomics to protect the individual and their relatives' well‐being. Participants understood that the genome contains a large amount of sensitive information about the individual and their relatives' health (eg predispositions, inherited conditions, the influence of environment and lifestyle), but also other intimate insights (eg personality, talents, behaviours, origins). Most citizens argued that genomic data are, therefore, more sensitive than other health data.

[DNA] says something fundamental about who I am. It contains more information about me than the result of a blood test, which only reveals specific diseases. DNA defines to a great extend who we are

Citizens felt like they were laying themselves bare when sharing their genomic data and feared that it could turn against them or their relatives, which would make them vulnerable. They referred in particular to weaknesses in health (eg diseases) or personality traits that are negatively considered by society (eg low tolerance to stress).

Citizen 1: There is a high level of distrust in this group. We distrust the secondary use of data after a person agreed to share their genome or their genomic data. […]

Citizen 2: We have the feeling that it will escape us and that there is some kind of danger

Therefore, citizens concluded that privacy should be one of the fundamental principles to guide the use of genomic information in society.

However, citizens pointed out that the legal protection of confidential data is worth nothing if individuals are not aware of their right to privacy, or act in a way that undermines it. Since they considered the genome as the most intimate part of individuals, citizens invoked several claim rights on their genomic data, including the right to control their access, sharing and uses. Respect for these right leads to privacy protection through increased autonomy, transparency and traceability. For instance, some citizens mentioned blockchain and notification technologies to inform them when someone would use their data for new purposes in research. Most participants thought that individuals have to safeguard their privacy to protect their autonomy, as the disclosure of genomic information could diminish the range of current and future choices in many fields, such as employment, mortgage credit or insurance.

### 3.2 I am more than my genome

Although citizens believed their genome reveals private information about their identity, they argued that individuals are more than their genetic make‐up. The genome does not tell the whole story about individuals who are socially and psychologically complex and should be treated and considered accordingly.

Addictions are intergenerational and are found in DNA, but we are more than that because we can wish to stop addictions

Moreover, citizens refused to believe in a deterministic conception of genomics as an individual's identity and health are determined by education, environment and lifestyle too. In other words, citizens feared being reduced to the information their genome may reveal and still worried that either themselves or others knowing about their genome could reduce their freedom and choices in life. Hence, citizens ardently defended that individuals should not be prisoners of their genetic make‐up and that DNA cannot be used as a quality label.

There is a risk that people say: ‘I have received my DNA screening results, so I am like this, this and this’. No, we are much more than that

Therefore, citizens asked for protections against any form of genetic discrimination, categorization and rejection. For example, employers should not use genetic test results of employees to redirect their careers according to their stress tolerance. To avoid situations like these, citizens recommended that third parties with the power to influence people's lives negatively—banks, insurance companies, employers and in some cases even the government—should not have access to genomic information. The fear of being prisoners of their genome also applied to private life since citizens recognized the psychological impact of DNA testing: one cannot ignore the information in one's genome once one knows it.

The sword of Damocles is hanging over her head. She has had no symptoms in all these years. It costs her dearly […] you know, the psychological aspect of feeling like it's going to start whenever you don't feel 100%. She says she would have rather not known. However, this is easy to say twenty years later because she is not sick; if she had been sick…

Besides anxiety, knowing one's genomic information can cause conflicts with relatives who may carry the inherited condition too. Consequently, citizens insisted on the right not to know and on the absence of societal pressure to adapt one's lifestyle to genetic test results. In brief, citizens claimed that individuals should have the right to an open future, whatever their genes may reveal.

### 3.3 Individual property or common good?

This question raised during the citizen forum resulted in one of the most significant conflicts of values among citizens. Those who viewed the genome within the framework of individual property argued that the individual is the right person to decide what can be done with their genomic information.

DNA is my property; it belongs to me mostly because it goes beyond health and a medical record. It is about identification. […] So I decide what I do with it

For some citizens, this included the right to sell their genomic data.

Everyone should be allowed to sell their [genomic] data. We do what we want with our data, like with our organs. However, it does not mean that it is a good thing to do

Conversely, the majority argued that, even though individuals are the owner of their genome, they are not free to use it in any way they want and they are still responsible for the consequences the dissemination of their genomic information may have on themselves and others. These citizens thought that legally permitting individuals to sell their genomic data would create a society focused on individual interests, instead of a more altruistic culture. They considered this immoral since one would make a profit from data one could freely share for scientific research and the good of society.

It is important to ensure that everyone remains the owner of their genome, but they should also develop a sense of common good. Even if my genome remains my property, it is still of interest to others. For this reason, we want to support a culture of giving instead of a culture of marketing

Following the same logic, the majority disapproved the individual's right to give third parties (eg insurers) access to their genomic information because this could lead to discrimination and inequality among the population.

Some citizens questioned the idea that the individual is the owner of their genomic information even more fundamentally by arguing that the genome is a common good. As individuals inherit their genes from their ancestors and pass them on to their children, individuals have the responsibility to consider their relatives when taking a genetic test.

Maybe this test will reveal genetic mutations that my brothers, sisters, and children may carry as well, with all the consequences it may have on them like needing treatments or testing, or whatever. So before getting them into that, perhaps I should ask their opinion, which would influence my decision to take the test or not […] because I would not make a decision I cannot own later

While some argued that the individual is the right person to decide whether to inform their relatives about test results or not, others proposed to allow doctors to break the medical secrecy in case of inherited, life‐threatening and curable diseases.

Additionally, most citizens pointed out that the genome binds humans since each individual could help others by sharing their genomic information for scientific research that enriches medical knowledge, improves the population's health (eg through personalized diagnosis, treatment and prevention) and therefore contributes to a fair society where everyone has an equal opportunity to a healthy life. They concluded that individuals have a moral responsibility to use their genomic information in a way that serves the general interest.

I feel that if I do the test for medical or other reasons, my results should be accessible to everybody, at a minimum to help my family […] or to support scientific progress. Thus, access for all. We must acknowledge that we are all connected, we are all Homo sapiens unless I am mistaken, we are part of the same big family. Therefore, it should be used for all, and not kept for oneself

While some citizens referred to the duties of beneficence and altruism and even referred to selfishness if someone does not act in the general interest, others tended to be more supportive of the principle of reciprocity (eg to link data sharing to reimbursement of the test). Whether an individual should be free to decide to share their genomic data remained a point of discussion. Some citizens suggested to leave the decision up to the individual, but to offer incentives for data sharing, to make it the default option or to raise awareness about the importance of data sharing to nudge citizens into acting in support of the common good. Still, all citizens except one argued that individuals should never be legally obliged to share their genomic data.

### 3.4 The genome implies uncertainty and fear

Citizens identified different levels of uncertainty in genomics, understood as ‘the conscious awareness of ignorance’. From their interactions with the experts, citizens understood that for most indications, genomic test results only show probabilities or risks, and often produce results of unknown significance as genomics is still a young science. Since the significance of so many genes still needs to be discovered, citizens were unsure about what kind of results scientists may find in their genome later.

Be careful. It is like opening Pandora's’ Box. You don't know what will come out

Consequently, citizens asked to be cautious with genomic testing and reminded that it should never be used as a quality label. This uncertainty that surrounds genomics resulted in ‘trust’ being one of the main concepts mentioned by participants.

Moreover, citizens were uncertain and suspicious about what research will make possible in the future since genomics is still in its infancy. They mainly feared the possible adverse uses of their genomic information, such as discrimination, eugenics or the creation of biological weapons. Regarding future generations, citizens worried about the potential reduction of human diversity through eugenics and genetic manipulation. Hence, citizens opposed programmes that aim to eliminate illness and disabilities systematically, or that could lead to the standardization of populations by enhancing certain features (eg intelligence).

The right to be yourself must be guaranteed; the right not to correspond to the cult of performance and perfection. This is related to our wish for a world with diversity, and to our fear of a world where everyone is conformed due to genomic manipulation and the like. We want to avoid that kind of world

Nevertheless, the majority of citizens supported the individual's right to use genomic technologies (eg prenatal screening, embryo selection, carrier screening) to avoid children with genetic diseases or disabilities, which may lead to the reduction of human diversity in the long run. A minority disapproved of this individual right because it discriminates and categorizes disabled and sick people, and may blame parents who choose to keep an at‐risk or a sick child. These citizens warned against the human tendency to ‘play God’.

It is the respect for human beings as they are, no matter if they are disabled or not […]. If we begin to select to that point, we take a role that is not ours, because nature already makes the selection. At a given moment, we may need people who carry this or that feature we considered as a disability, or people who don't have the perfect genome, but who resist one specific disease others don't resist. It is how nature has always worked

While the present results focus on the negative aspects of genomics according to the citizen panel, they recognized the tremendous potential for good inherent in genomics too. They supported genomic testing to help prevent diseases by detecting predispositions and inherited conditions; they encouraged genomic data sharing to develop better treatments and improve medical knowledge, which contributes to a fair society where everyone has an equal opportunity to a healthy life. In short, genomics brings many hopes and fears simultaneously. To preserve the support of the public and avoid their fear becoming a reality, the citizen panel demanded a clear legal and ethical framework that inspires trust by using a soft precautionary principle to guide the governance of genomic data.

## 4 DISCUSSION

The four conceptions of the genome from citizens illustrate the ambiguity and complexity of the ways citizens approach genomics. These conceptions lead to various fundamental norms and values concerning the use of genomic information in society. We argue that it is important to understand this framework of different conceptions to be able to interact with citizens about their values and opinions.

Firstly, citizens considered that their genome is part of their intimacy and that the use of their genomic information entails vulnerability for themselves and their relatives. Hence, citizens expected the government to protect their privacy on the one hand by increasing individuals’ empowerment and control (eg a dynamic informed consent, including the right to revisit it in case the purposes of the uses change) and on the other hand by providing institutional oversight on data security and limited access to genomic data. In turn, citizens should act responsibly to safeguard their privacy, for instance, by not giving third parties such as employers, insurers or banks access to their genomic data. Secondly, as the genome does not tell the whole story about individuals, citizens asked protection against any form of genetic categorization and discrimination to protect their freedom of choice and their right to an open future. This right to an open future also included the right not to know one's genomic information. Thirdly, citizens viewed the genome simultaneously as an individual property and a common good, which implies both the autonomy in decision making and responsibilities towards others who may be affected by or benefit from the use of one's genomic information. Regarding data sharing, it meant for citizens that this decision should be left to individuals, but might be coupled with nudging from society, such as making data sharing the default option with the possibility to withdraw, or educating the public and raising their awareness about its benefits for public health. According to citizens, data sharing supposes balancing one's interests with the interests of others, either under the principle of reciprocity (eg link data sharing to reimbursement of the test), altruism or solidarity (everyone has a moral responsibility to share their data for the good of society). Finally, the genome causes uncertainty and fears to citizens who, therefore, needed to trust any person or institution who uses their genomic data, and demanded that the precautionary principle steers any framework in genomics. From the citizens' perspective, this implies a clear legal and ethical framework—whether through laws, regulations, guidelines or recommendations—specifically on the use of genomic information, which should guarantee transparency and traceability in those uses (eg blockchain and notification technologies). Yet, this framework should not be too restrictive with the risk of limiting scientific progress, but rather adaptive and flexible.

The conceptions of the genome from this study align with findings of previous public engagement studies, both qualitative and quantitative, on ELSI in genomics. Previous literature showed that participants consider their genome to be part of their private life as it enables to identify them, contains a lot of personal data about them and their relatives, and because the dissemination of their genomic information could harm them.^3^, ^4^, ^5^, ^6^, ^7^, ^8^, ^20^, ^21^, ^22^, ^23^, ^24^, ^25^, ^27^ Discrimination by insurances, banks or employers constitutes one of the biggest concerns mentioned by the public as well.^3^, ^4^, ^5^, ^8^, ^9^, ^10^, ^11^, ^12^, ^13^, ^14^, ^15^, ^16^, ^17^, ^18^, ^19^, ^20^, ^21^, ^23^, ^24^, ^26^, ^27^, ^28^ However, several studies reported that some participants put privacy into perspective, either by being ready to trade off complete confidentiality against potential benefits for themselves and others or by adopting a privacy‐resignation stand in this society that collects a large amount of personal data every day.^3^, ^4^, ^5^, ^6^, ^7^, ^8^, ^23^, ^24^, ^25^, ^26^, ^27^ Some citizens from the Belgian forum also balanced their right of privacy with their relatives' health (eg breaking the medical secrecy in case of inherited, life‐threatening and curable diseases) and with the common good (eg sharing their genomic data to improve the population's health).

Because the citizen panel regarded their genome as their most intimate part, they argued that genomic information is exceptional or different from other medical information, which is called genetic exceptionalism. The exceptionalist view has been highlighted as well in other recent studies. In the survey ‘Your DNA, Your Say’, 52% of the participants from the USA, UK, Canada and Australia held this point of view.^25^ German participants holding an exceptionalist position argued that the genome is unique, is the most personal information of individuals and belongs to them.^29^ Furthermore, a recent qualitative study indicated that participants consider genomic data as exceptional not because of an inherent difference, but merely because of its uses outside of health care and its familial implications.^8^

Finally, the citizen panel defined the genome both as an individual property and a common good shared with relatives and humanity by extension. This ambivalent conception of the genome has been noted in previous studies, where participants understood the many individual interests and risks in genomics, but also that their genomic information always implicates other people. For example, when asked about their motivation to participate in genomic research, participants from several studies mentioned both benefits for their health and the health of their relatives, their descendants and the global population.^8^, ^9^, ^10^, ^11^, ^12^, ^13^, ^14^, ^15^, ^16^, ^17^, ^18^, ^19^, ^20^, ^21^, ^22^, ^23^, ^30^ While some participants in earlier studies highlighted the perception of the genome as a common good by referring to altruism, solidarity and even responsibility as citizens and human beings,^8^, ^9^, ^10^, ^11^, ^12^, ^13^, ^14^, ^15^, ^16^, ^17^, ^18^, ^19^, ^20^, ^21^, ^22^, ^23^, ^24^, ^25^, ^26^, ^27^, ^28^, ^29^, ^30^ other participants emphasized an individualistic understanding of the genome by referring to concepts such as reciprocity, respect for an individual's autonomy,^29^ informed consent,^7^, ^8^ transparency and being up‐to‐date on the uses of their data and new genetic results.^20^, ^21^, ^22^, ^23^, ^24^, ^25^, ^26^, ^27^, ^30^

Although a consensus might not always be reached on how genomic information should be used in society because tensions of values persist among participants, it is still necessary to look at those values and norms behind citizens' opinions because they indicate which uses of genomic information are supported. Nevertheless, participants were aware that it is up to capable legislators, experts and stakeholders to further develop their recommendations into practical tools for the real world. These parties will have to take the convictions and values of citizens into account as one of several valuable sources to build a just normative and legislative framework for the implementation of genomics.

## 5 CONCLUSION

Without public trust and support, genomic medicine will not be able to fulfil its commitment to personalize care and improve public health. The Belgian citizen forum shows that the public can make meaningful contributions to a complex field such as ELSI in genomics. Health decision makers, experts and stakeholders in the field of genomics should understand how citizens consider the human genome and the values they assign to it as these inform which uses of genomic information are supported in society. Diversity in Belgian citizens’ conceptions of the genome reveals remaining conflicts of values—for instance, reciprocity vs altruism in data sharing. But mostly, these conceptions articulate shared values where individual and common interests coexist, such as promoting solidarity by sharing genomic data for the good of society (eg genomic medicine and scientific research) while protecting individuals’ privacy, their right to an open future and avoiding genetic discrimination. We conclude that continued citizen involvement in the governance of genomic information is vital to ensure that societal norms and values guide the technology and not the other way around.

## CONFLICT OF INTEREST

All authors have no conflict of interest to declare.

## Data Availability

The data that support the findings of this study are available from the corresponding author upon reasonable request.

## References

[1] Rehm HL . Evolving health care through personal genomics. Nat Rev Genet. 2017;18(4):259‐267. 10.1038/nrg.2016.162 28138143 PMC6517837

[2] Stark Z , Dolman L , Manolio TA , et al. Integrating genomics into healthcare: a global responsibility. Am J Hum Genet. 2019;104(1):13‐20. 10.1016/j.ajhg.2018.11.014 30609404 PMC6323624

[3] Dheensa S , Lucassen A , Fenwick A . Fostering trust in healthcare: participants' experiences, views, and concerns about the 100,000 genomes project. Eur J Med Genet. 2019;62(5):335‐341. 10.1016/j.ejmg.2018.11.024 30503854

[4] Middleton A , Morley KI , Bragin E , et al. Attitudes of nearly 7000 health professionals, genomic researchers and publics toward the return of incidental results from sequencing research. Eur J Med Genet. 2016;24(1):21‐29. 10.1038/ejhg.2015.58 PMC4795240 25920556

[5] Middleton A , Milne R , Thorogood A , et al. Attitudes of publics who are unwilling to donate DNA data for research. Eur J Med Genet. 2019;62(5):316‐323. 10.1016/j.ejmg.2018.11.014 30476628 PMC6582635

[6] Milne R , Morley KI , Howard HC , et al. Trust in genomic data sharing among members of the general public in the UK, USA, Canada and Australia. Human Genetics. 2019;138(11–12):1237‐1246. 10.1007/s00439-019-02062-0 31531740 PMC6874520

[7] Comité Consultatif national d’éthique . Rapport de synthèse du Comité Consultatif national d’éthique: opinions du Comité citoyen. EDP Sciences; June 2018. https://etatsgenerauxdelabioethique.fr/media/default/0001/01/cd55c2a6be2d25e9646bc0d9f28ca25e412ee3d4.pdf. Accessed June 29, 2020.

[8] Castell S , Bukowski G , McAneney H . A public dialogue on genomic medicine: time for a new social contract?. Ipsos Mori; April 2019. https://www.ipsos.com/sites/default/files/ct/publication/documents/2019‐04/public‐dialogue‐on‐genomic‐medicine‐full‐report.pdf. Accessed June 29, 2020.

[9] Soulier A , Niemiec E , Howard HC , et al. Ethical analysis of human genetics and genomics. SIENNA; August 2019. https://ec.europa.eu/research/participants/documents/downloadPublic?documentIds=080166e5c70bb81d&appId=PPGMS. Accessed July 30, 2020.

[10] Bohman J , Chambers S , Christiano T , et al. A systemic approach to deliberative democracy. In: Williams M , ed. Jane Mansbridge: Participation, deliberation, legitimate coercion. Oxfordshire: Taylor & Francis; 2018:175‐188.

[11] Lafont C . Deliberation, participation, and democratic legitimacy: should deliberative mini‐publics shape public policy? J Political Philos. 2015;23(1):40‐63. 10.1111/jopp.12031

[12] Felicetti A . Citizen forums in the deliberative system. Democratic Theory. 2014;1(2):95‐103. 10.3167/dt.2014.010210

[13] King Baudouin Foundation , Sciensano . La connaissance du génome influence les soins de santé – Les citoyens demandent une politique pour l'avenir. Brussels: King Baudouin Foundation; 2019. https://www.kbs‐frb.be/fr/Activities/Publications/2019/20190717PP. Accessed June 29, 2020.

[14] King Baudouin Foundation , Sciensano . Mon ADN, tous concernés ? Débat de société sur l’utilisation des données du génome dans le cadre des soins de santé. Brussels: King Baudouin Foundation; 2018. https://www.kbs‐frb.be/fr/Activities/Publications/2018/20180704PP. Accessed June 29, 2020.

[15] Marien S , Felicetti A . Citizen forum on the use of genome information in health care: an assessment of the quality of the process. Leuven: KU Leuven; 2019. https://soc.kuleuven.be/centre‐for‐political‐research/demoinno/files/citizens‐reporten‐executive‐reportnl‐fr.pdf. Accessed June 29, 2020.

[16] King Baudouin Foundation , Sciensano . Mon ADN: tous concernés? L’avis des citoyens sur l’utilisation des données du génome dans les soins de santé. Brussels: King Baudouin Foundation; 2019. https://www.kbs‐frb.be/fr/Activities/Publications/2019/20190225PP2. Accessed June 29, 2020.

[17] King Baudouin Foundation , Sciensano . The use of genome information in health care: ethical, legal and societal issues – Issue framing workshop report. Brussels: King Baudouin Foundation; 2018. https://www.kbs‐frb.be/nl/Activities/Publications/2018/20180712PP. Accessed June 29, 2020.

[18] Mayeur C , van Hoof W . My DNA, everybody’s business? Qualitative analysis of the Belgian citizen forum on the use of genomic information. Brussels: Sciensano; 2020. https://www.e‐cancer.be/fr/final‐report‐my‐dna‐everybodys‐business‐qualitative‐analysis‐belgian‐citizen‐forum‐use‐genomic. Accessed June 29, 2020.

[19] Braun V , Clarke V . Using thematic analysis in psychology. Qual Res Psychol. 2006;3(2):77‐101. 10.1191/1478088706qp063oa

[20] Human Genetics Commission . Public attitudes to human genetic information: people's panel quantitative study conducted for the human genetics commission. December 2000. https://health.ucdavis.edu/biorepositories/pdfs/genomics‐biobank/Public‐attitudes‐to‐human‐genetic‐information.pdf. Accessed June 29, 2020.

[21] Lemke AA , Wolf WA , Hebert‐Beirne J , Smith ME . Public and biobank participant attitudes toward genetic research participation and data sharing. Public Health Genomics. 2010;13(6):368‐377. 10.1159/000276767 20805700 PMC2951726

[22] Kaufman D , Murphy J , Erby L , Hudson K , Scott J . Veterans' attitudes regarding a database for genomic research. Genet Med. 2009;11(5):329‐337. 10.1097/GIM.0b013e31819994f8 19346960

[23] Godard B , Marshall J , Laberge C . Community engagement in genetic research: results of the first public consultation for the Quebec CARTaGENE project. Community Genetics. 2007;10(3):147‐158. 10.1159/000101756 17575459

[24] Wong ML , Chia KS , Wee S , et al. Concerns over participation in genetic research among Malay‐Muslims, Chinese and Indians in Singapore: a focus group study. Community Genetics. 2004;7(1):44‐54. 10.1159/000080303 15475670

[25] Middleton A , Milne R , Howard H , et al. Members of the public in the USA, UK, Canada and Australia expressing genetic exceptionalism say they are more willing to donate genomic data. Eur J Hum Genet. 2020;28:424‐434. 10.1038/s41431-019-0550-y 31784701 PMC7080803

[26] Voss G . Report to the Human Genetics Commission on public attitudes to the uses of human genetic information. January 2000. https://www.researchgate.net/profile/Georgina_Voss2/publication/238768948_Report_to_the_0Human_Genetics_Commission_on_Public_Attitudes_to_the_Uses_of_Human_Genetic_Information/links/542201540cf2a39f4af76882/Report‐to‐the‐Human‐Genetics‐Commission‐on‐Public‐Attitudes‐to‐the‐Uses‐of‐Human‐Genetic‐Information.pdf. Accessed June 29, 2020.

[27] Hobbs A , Starkbaum J , Gottweis U , Wichmann HE , Gottweis H . The privacy‐reciprocity connection in biobanking: comparing German with UK strategies. Public Health Genomics. 2012;15(5):272‐284. 10.1159/000336671 22722691

[28] Comité Consultatif national d’éthique . Synthèse cartographique de la consultation publique en ligne. Cap Collectif; June 2018. https://etatsgenerauxdelabioethique.fr/media/default/0001/01/0c9bcd708ab2eec9f70d67459265503068000f49.pdf. Accessed June 29, 2020.

[29] Voigt TH , Holtz V , Niemiec E , Howard HC , Middleton A , Prainsack B . Willingness to donate genomic and other medical data: results from Germany [published online ahead of print April 1. Eur J Hum Genet. 2020;28(8):1000‐1009. 10.1038/s41431-020-0611-2 32238912 PMC7381614

[30] Michie M , Henderson G , Garrett J , Corbie‐Smith G . "If I could in a small way help": motivations for and beliefs about sample donation for genetic research. J Empiri Res on Human Res Ethics. 2011;6(2):57‐70. 10.1525/jer.2011.6.2.57 PMC3313647 21680977

